# Carbohydrate functionalization of silver nanoparticles modulates cytotoxicity and cellular uptake

**DOI:** 10.1186/s12951-014-0059-z

**Published:** 2014-12-19

**Authors:** David C Kennedy, Guillermo Orts-Gil, Chian-Hui Lai, Larissa Müller, Andrea Haase, Andreas Luch, Peter H Seeberger

**Affiliations:** Department of Biomolecular Systems, Max Planck Institute of Colloids and Interfaces (MPIKG), 14476 Potsdam, Germany; Division 1.1 Inorganic Trace Analysis, Federal Institute for Materials Research and Testing (BAM), Richard-Willstätter-Straße 11, 12489 Berlin, Germany; Departments Chemical and Product Safety, German Federal Institute for Risk Assessment (BfR), 10589 Berlin, Germany; Institute for Chemistry and Biochemistry, Free University Berlin, Arnimallee 22, 14195 Berlin, Germany; National Research Council Canada (CNRC), 100 Sussex Drive, Ottawa, Ontario Canada

**Keywords:** Silver, Nanoparticles, Carbohydrates, Nanotoxicology, Bio-interfaces

## Abstract

**Background:**

Increasing use of silver nanoparticles (Ag-NPs) in various products is resulting in a greater likelihood of human exposure to these materials. Nevertheless, little is still known about the influence of carbohydrates on the toxicity and cellular uptake of nanoparticles.

**Methods:**

Ag-NPs functionalized with three different monosaccharides and ethylene glycol were synthesized and characterised. Oxidative stress and toxicity was evaluated by protein carbonylation and MTT assay, respectively. Cellular uptake was evaluated by confocal microscopy and ICP-MS.

**Results:**

Ag-NPs coated with galactose and mannose were considerably less toxic to neuronal-like cells and hepatocytes compared to particles functionalized by glucose, ethylene glycol or citrate. Toxicity correlated to oxidative stress but not to cellular uptake.

**Conclusions:**

Carbohydrate coating on silver nanoparticles modulates both oxidative stress and cellular uptake, but mainly the first has an impact on toxicity. These findings provide new perspectives on modulating the bioactivity of Ag-NPs by using carbohydrates.

**Electronic supplementary material:**

The online version of this article (doi:10.1186/s12951-014-0059-z) contains supplementary material, which is available to authorized users.

## Introduction

Nanoparticles are playing an increasing role in the development of novel diagnosis methods and in the advanced design of drug delivery systems [1,2]. Silver nanoparticles (Ag-NPs) in particular, show excellent anti-microbial properties and therefore are rapidly being incorporated into a wide array of consumer products such as textiles, cosmetics or packaging materials, increasing the likelihood of human and environmental exposure [3,4]. Moreover, due to their optical properties Ag-NPs are attracting more attention in the fields of biological and chemical sensors [5]. However, Ag-NPs exist in variety of different sizes and shapes but also, very important, with different coatings. Recently, among surface coatings there is an increasing interest in using carbohydrates as biomimetic functional molecules on the surface of nanoparticles [2,6-8] for the diagnosis and treatment, for instance, of brain diseases like glioma and stroke [9,10]. Glycan functionalised NPs offer several advantages: (i) their synthesis can be performed under biomimetic conditions resulting later on in nanoparticles without traces of chemicals responsible for adverse cellular responses. (ii) the carbohydrates on the surface can serve as targeting molecules and trigger cellular uptake via specific receptors or mediate specific cellular responses [10]. Concurrently, the importance of carbohydrates in cellular signalling and in the regulation of cellular processes continues to emerge [11]. The inherently weak interactions between carbohydrates and proteins or other biomolecules makes these interactions difficult to study. However, because these interactions tend to be multivalent in nature, the use of nanoparticles to mimic the multivalent presentation of carbohydrates found on biomolecular surfaces make carbohydrate-functionalized nanoparticles important systems to study [12].

Several factors like surface charge and particle size can contribute to the selective binding and uptake of nanomaterials [13,14]. In addition to labelling with a targeting molecule, nanoparticles can induce multivalent effects by clustering antigens on the surface of the particle. Thereby, the binding of relatively weak targeting agents can be enhanced.

Nevertheless, despite the importance of carbohydrates in biology and the vast array of literature on functionalized nanomaterials, little is known about the effects of carbohydrates on the uptake and toxicity of nanoparticles by different type of cells. Although it has been reported that polysaccharides can reduce the toxicity of silver nanoparticles [15] less is known about the influence of monosaccaharides [16] thus, the different results are difficult to rationalise.

Moreover, as pointed out by Johnston et al. [17], the increasing importance of Ag-NPs in the development of novel consumer materials intended for human exposure requires more in depth studies on toxicity mechanisms, as well as, on how silver particles interact with biological molecules and how different surface modifications can be used to reduce or eliminate possible toxic effects.

Here, we discuss the toxicity and the cellular uptake of different silver nanoparticles functionalized with citrate, three different monosaccharides as well as ethylene glycol on two different cell lines. It was found that toxicity correlates with oxidative stress rather than with cellular uptake.

## Experimental

### Materials

Silver nitrate, sodium citrate, D-glucose, D-mannose, D-galactose and ethylenglycol with MW = 200 (EG-3) with purity >99% were purchased from Sigma-Aldrich.

### Synthesis of silver nanoparticles

Citrate-capped silver nanoparticles were synthesized using a standard method [18]: A solution of AgNO_3_ (10^−3^ M) in deionized water was heated until boiling point. Then, sodium citrate solution in water was added dropwise. The color of the solution slowly turned into gray-yellow after few minutes, indicating the formation of nanoparticles. Heating was continued for an additional 10 min. The solution was then cooled to room temperature and stored in dark before functionalization.

### Synthesis of carbohydrate ligands

Thiol-functionalized carbohydrates were synthesized using the following process: glucose, galactose and mannose were each reacted with thiopropionic acid to form the corresponding glycoside in approximately 50% yield. The products were isolated as DMAP-H+ salts. Solutions of these thiolate salts were then directly added to solutions of silver nanoparticles to prepare the carbohydrate functionalized particles.

### Functionalization of nanoparticles

**Figure 1:**
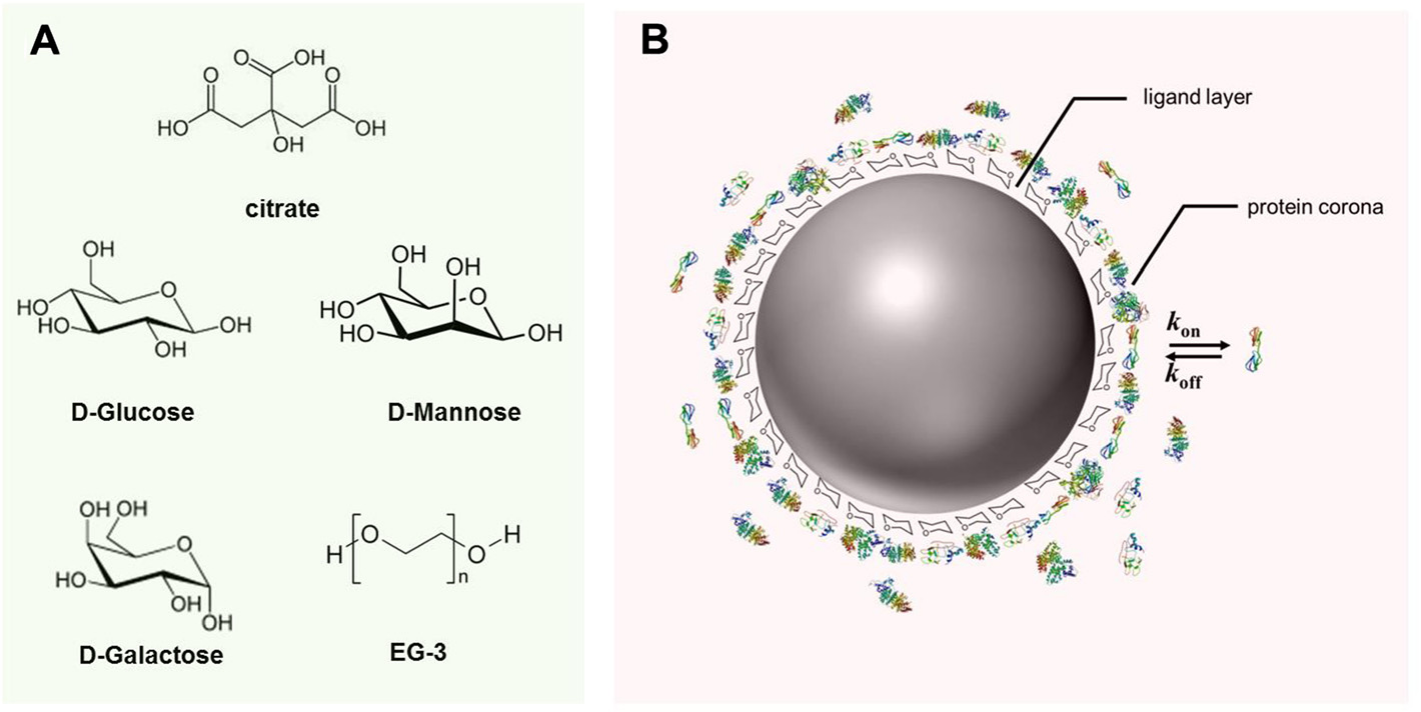
**Prepared nanoparticles. (A)** Different biomolecules on the surface of prepared silver nanoparticles (linker not shown); **(B)** in cell culture media the nanoparticle-water interface is composed of ligands and ions but also of proteins, the so-called protein corona.

To a glass vial charged with a stir bar was added 1 mL of citrate capper silver nanoparticles (1 nM) and 60 μL of a 2 mM stock solution of the corresponding ligand. The solutions were stirred for 3 h at which time each solution was transferred to a 1.5 mL Eppendorf tube and centrifuged for 10 min at 8000 rpm. The supernatant was discarded and the pellet was resuspended in 1 mL of H_2_O. These nanoparticle solutions were then used for subsequent reactions and analyses. Scheme of synthesized nanoparticles is shown in Figure 1.

### Nanoparticles characterization

#### Dynamic light scattering (DLS)

Most of DLS measurements were carried out at 37°C (to simulate physiological temperature) by use of a Malvern Zeta Nanosizer in water and in cell culture medium (DMEM + 10% FCS). This instrument operates at 4-mW He-Ne laser power, scattering angle of 173° and a wavelength of 633 nm. The intensity correlation functions were fitted by the method of cumulants and by using the Non-Negative Least Squares algorithms (NNLS) included in the Zeta Nanosizer software. The zeta potentials of the samples were obtained from laser Doppler electrophoresis, converting electrophoretic mobilities to zeta potentials. Each sample was prepared in triplicate and measured six times. Experiments consisted of 60 runs per measurement.

#### Transmission electron microscopy (TEM) and energy dispersive X-ray spectroscopy (EDS)

TEM investigations were performed on a Jeol JEM 2200-FS operating at 200 kV. At high magnification, the in-column Ω-filter was used to improve the contrast. Samples were prepared by immersion of grids of S-160-3 type (Cu coated with carbon film, Plano GmbH) in a small volume (0.5 mL) of solutions followed by solvent evaporation in a dust-protected atmosphere. Particle size distributions were obtained by analysing at least 200 NPs from TEM images using ImageJ software [19]. Energy dispersive X-Ray analysis (EDX) was performed in STEM mode using a spot size between 0.5 and 1.5 nm.

#### Sugar quantification

Sugar densities were evaluated for Glu, Gal and Mann by a previously reported method [2,7]. Briefly, Glu-Ag-NPs, Gal-Ag-NPs and Mann-Ag-NPs were dispersed in deionized water (0.5 mL) in an ice bath. A freshly prepared 0.5% (w/w) solution of anthrone in sulfuric acid (1 mL) was added slowly to this solution. The resulting solution was gently mixed and heated to 80°C for 10 min. The absorption of the solution was measured at 620 nm and compared with those that were obtained from a standard curve to determine the amount of sugars on the Ag-NP surface.

### Cell culture

Neuro-2A and HepG2 cells (American Tissue Culture Center) were each grown in Dulbecco’s modified Eagle’s medium supplemented with 10% fetal bovine serum (PAN-Biotech), 1% penicillin-streptomycin (50 μg/mL, PAN-Biotech), and l-glutamine (2 mM, PAN-Biotech), under standard culture conditions (37°C, 5% CO2).

### MTT assay

Cells were seeded into wells in a 96-well plate (1 × 105 cells/mL, 100 μL per well) to cover a 9 × 6 grid, filling 54 wells. Remaining wells were filled with 200 μL of PBS. After 24 hours, 100 μL volumes of dilutions of nanoparticles in water spanning from 1 nM to 0.01 nM were added to the seeded wells (final concentrations spanning 5 pM to 500 pM). For each functionalized particle, eight dilutions were prepared and for each dilution six replicates were performed. In the remaining 6 wells, 100 μL of PBS was added as a control. Cells were then incubated with complexes for 72 h. After 72 h, 50 μL of a PBS solution of MTT (2.5 mg/mL) was added to each well and then incubated for 3 h. After 3 h, media was aspirated from all wells, leaving purple formazan crystals in those wells with viable cells. To each well, 150 μL of DMSO was added. Plates were then agitated for 10 s and analyzed using a plate reader (NanoQuant Infinite M200 instrument by Tecan Group Ltd.) to determine the absorbance of each well at 570 nm. This reading divided by the average from the reading of the six control wells was plotted to determine the IC50 value of each complex for each cell line.

### Analysis of protein carbonylation as a read-out for intracellular oxidative stress development

HepG2 were seeded in 6-well plates and treated with nanoparticles (final concentrations 2.5 pM and 5pM) for 6 h (induction of protein carbonylation). Cells were washed with PBS three times and lysed by adding a modified RIPA buffer (50 mM Tris/HCl pH 7.4; 150 mM NaCl, 1 mM EDTA, 1% Igepal, 0.25% Na-deoxycholate). Protein concentrations were determined via Bradford assay according to manufacturer instructions (BioRad, München, Germany). For detection of protein carbonyls OxyBlot kit (Millipore, Schwalbach, Germany) was used according to manufacturer instructions. Briefly, protein carbonyls are labeled by adding 2,4-dinitrophenyl (DNP) hydrazine, which becomes covalently attached as DNP hydrazone and can be detected with the respective DNP antibody. SDS-PAGE was performed according to standard protocols. Gels were transferred onto nitrocellulose membranes with a semidry blotting system. Tubulin antibody was obtained from Abcam (Abcam, Cambridge, UK) and used as a loading control. Images were obtained with GelDoc system (BioRad, München, Germany) and quantified with ImageLab (BioRad, München, Germany). The assay was repeated in three independent experiments and results were statistically evaluated.

### Confocal microscopy

For imaging, cells were grown on cover slips seeded in 6-well plates. Sterilized cover slips were placed in each well followed by addition of cell suspensions (1 × 10^5^ cells/mL, 2 mL per well). After 24 h, cells were treated with either CuSO_4_ or CuHis to a final concentration of 25 μM. Cells were incubated with copper complexes for 1, 3, 24 or 72 h at which times the media was removed and cells were fixed by adding 1 mL of fixing solution (3.7% formaldehyde, 4% glucose in PBS). The fixing solution was then removed and 1 mL of PBS was added to cells in each well. To each sample, 4 μL of anti-PRP antibody (Abcam EP1802Y – rabbit monoclonal antibody against prion protein) were then added to the PBS and the cells were placed in the fridge to be treated for 12 h at 4°C. After 12 h, the PBS was removed, and cells were rinsed 3 times with 1 mL PBS. Cells were then treated with 1 mL PBS and 4 μL of a secondary Goat anti-Rabbit IgG – FITC (Invitrogen) and covered with foil. Cells were left at room temperature for 3 h and then treated with DAPI (3 μL Invitrogen). DAPI was added to each well and the plates were then covered with foil again and left at room temperature for 20 min. Finally, the PBS was aspirated, and cells were rinsed 3 times with 1 mL PBS. Cover slips were then removed from the wells. To prepare slides, PBS (20 μL) was added to the surface of each microscope slide and then the removed coverslips were inverted and placed on the PBS. Coverslips were then sealed using nail polish and dried in the dark for 10 min. Slides were imaged using a confocal laser scanning microscope (LSM 700, Zeiss). Z-stack plots (1 micron thick layers) were taken for 6 unique cell clusters from each sample. Stacks were compressed into two-dimensional images using ImageJ software to create a single image showing the entire cell surface. This image was then analyzed using voxel analysis to determine the number of fluorescently labeled pixels, and thus, the level of prion protein at the cell surface. Changes in surface expression and localization were noted and reported.

### ICP-MS

Cell samples were digested in 100 μL nitric acid and stored at −20°C until analysis. A 50 μL volume of the samples was diluted 1:10000 with 3.5% nitric acid for analysing the cellular uptake of NPs. Lanthanum (10 ppb) was added as internal standard. An external calibration series from 0.5 ppb to 50 ppb was prepared using a silver standard solution. A sample volume of 3 mL was needed for analysis. For this purpose an ICAP-Q (Thermo Fisher Scientific GmbH, Dreieich, Germany) was connected to a concentric nebulizer with a cyclone spray chamber. The working parameters are in Additional file 1.

## Results

**Figure 2:**
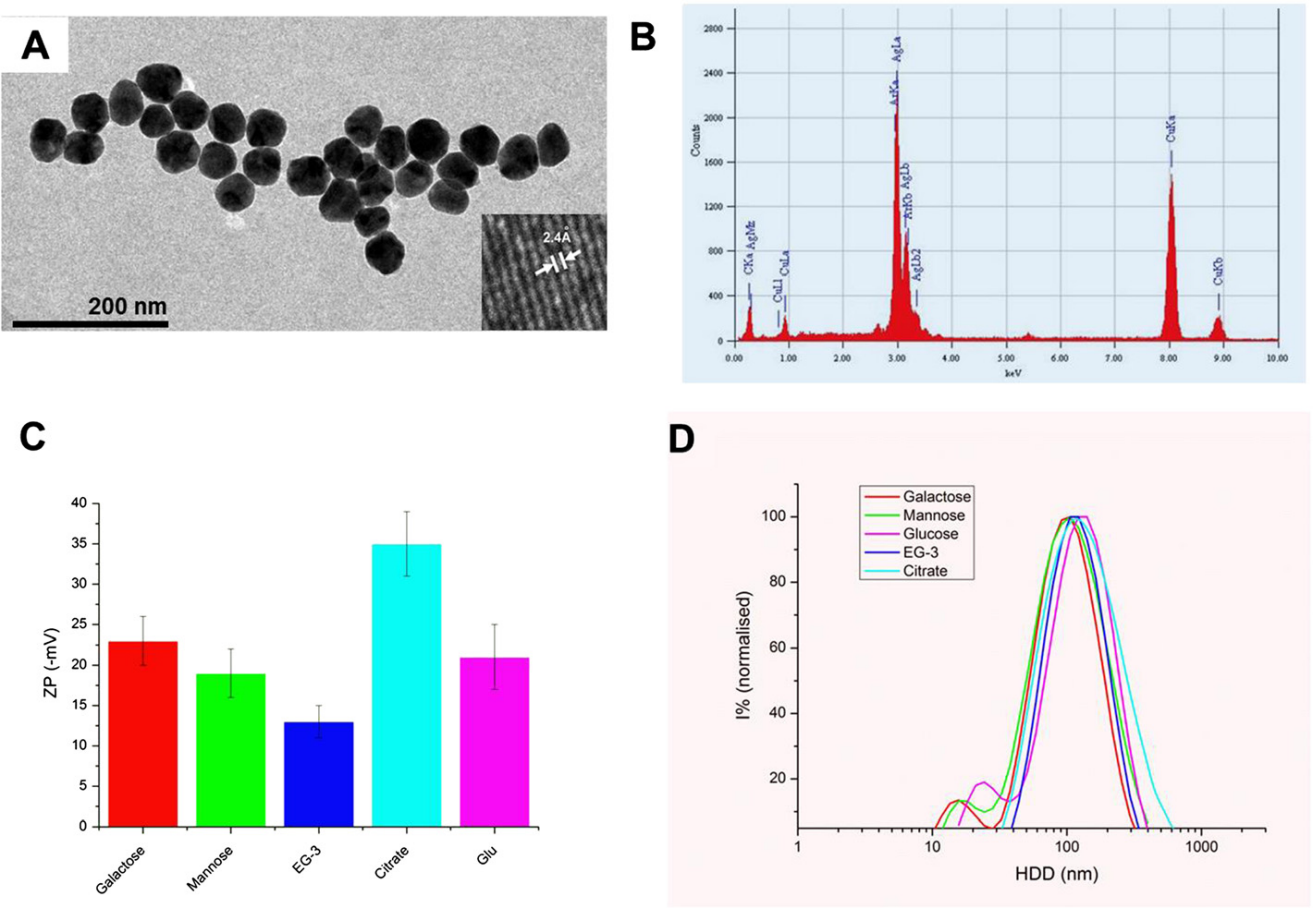
**Physico-chemical characterization of nanoparticles. (A)** TEM. Inset corresponds to high resolution image showing d lattices; **(B)** Energy dispersive X-ray analysis showing Ag but no impurities; **(C)** zeta potential of different prepared samples; **(D)** DLS of samples in cell culture medium showing well-dispersed nanoparticles.

All nanoparticles were characterized by TEM, DLS, ZP and EDX (Figure 2). Particle sizes computed from statistical analysis of TEM images were around 54 nm (Figure 2-A and Additional File 1). EDX confirms the absence of impurities (Figure 2-B). DLS of particles in water after 24 h show some degree of agglomeration while particles in cell culture media were more stable probably due to the formation of a protein corona (Figure 1-D and Additional File 1). This in good agreement with findings from Kittler et al. [20] and excludes, in this case, agglomeration as a factor affecting toxicity in cell culture medium [21,22]. ZP shows a change in surface charge for functionalized nanoparticles compared with citrate silver in good agreement with expected values for glycosylated nanoparticles [10] (Figure 2-C). Amount of sugar on nanoparticles was determined using the anthorne/H_2_SO_4_ method in a similar way as in our previous contribution [23] (see Additional file 1). Values found were between 3.2 and 3.9 molecules sugar/nm^2^.

**Figure 3:**
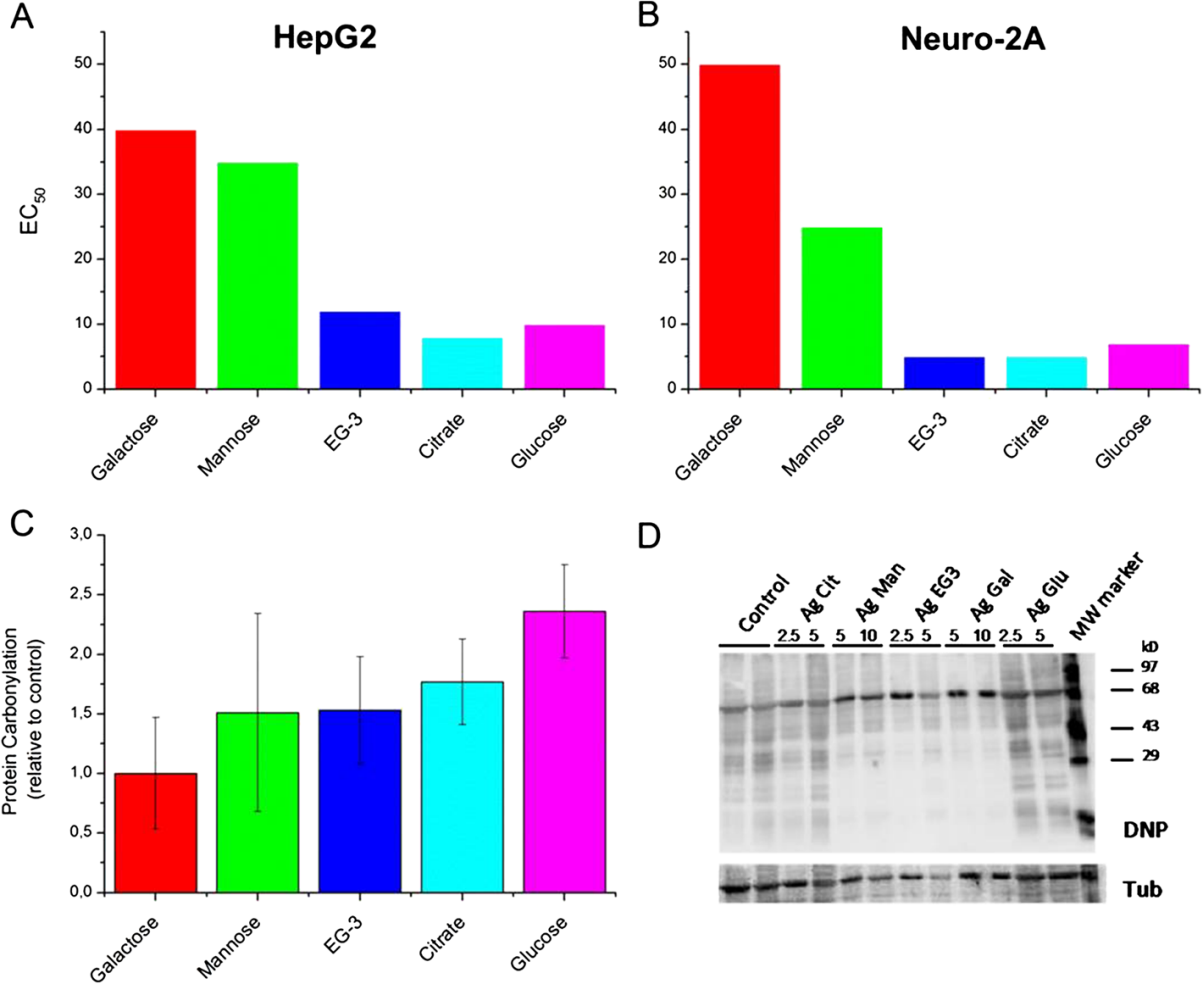
**Toxicity results in vitro. (A)** EC50 values from MTT assay using silver nanoparticles with different coatings and HepG2 cells; **(B)** Analogous with Neuro-2 cells; **(C)** Detection of oxidative stress from Ag-NPs (concentration 5 pM) via formation of protein carbonyls incubated with HepG2 cells. **(D)** Protein carbonyls were detected at different concentrations (2.5, 5, 10 pM) as (DNP) hydrazone adducts via immunoblots with a DNP antibody.

The toxicity of the functionalized silver nanoparticles was tested against two cell lines, a neuronal-like cell line (Neuro-2A) and a hepatocyte cell line (HepG2) by using an MTT assay (Figure 3-A and B). Here, a clear influence of the coating on the toxicity of the particles was observed. While particles functionalized with EG-3, glucose and citrate coated nanoparticles show a similar toxicity, galactose and mannose functionalized nanoparticles were significant less toxic towards both cell lines.

In order to elucidate the mechanism leading to observed coating-dependent toxicities we analyzed the formation of protein carbonyls as an indirect read-out for the oxidative stress inducing activity of nanoparticles. Proteins can become carbonylated either as a direct or an indirect consequence of reactive oxygen species (ROS) formation. Experiments were performed at particles concentrations 2.5 pM and 5 pM (see Additional file 1). At both concentrations a strong correlation between carbonyls formation and EC_50_ values can be observed (Figure 3-C), suggesting that the toxicity is mainly caused by oxidative stress related to ROS formation.

This may be related to ion release as has already been shown that silver ions can trigger oxidative stress. Many authors have argued that in fact the toxicity of nanosilver is only caused by the ionic form [24]. Therefore, in our case this could mean that either the different types of nanosilver are related to different release rates of ionic silver from the various different coated NP. Dissolution of silver nanoparticles can vary from less than 10% to up to nearly 100%, depending on the coating [25]. Since production of protein carbonyls rather simulates intracellular oxidative stress, the release of silver ions in cell culture media was also measured by ICP-MS in order to also evaluate potential extracellular oxidative stress (see Additional file 1). Nevertheless, no free silver ions were detected in the supernatant of cell culture media, probably due to precipitation of ionic silver in form of AgCl and protein complexes. Therefore, under the studied conditions, intracellular release of silver ions may be the only responsible for cellular damage.

**Figure 4:**
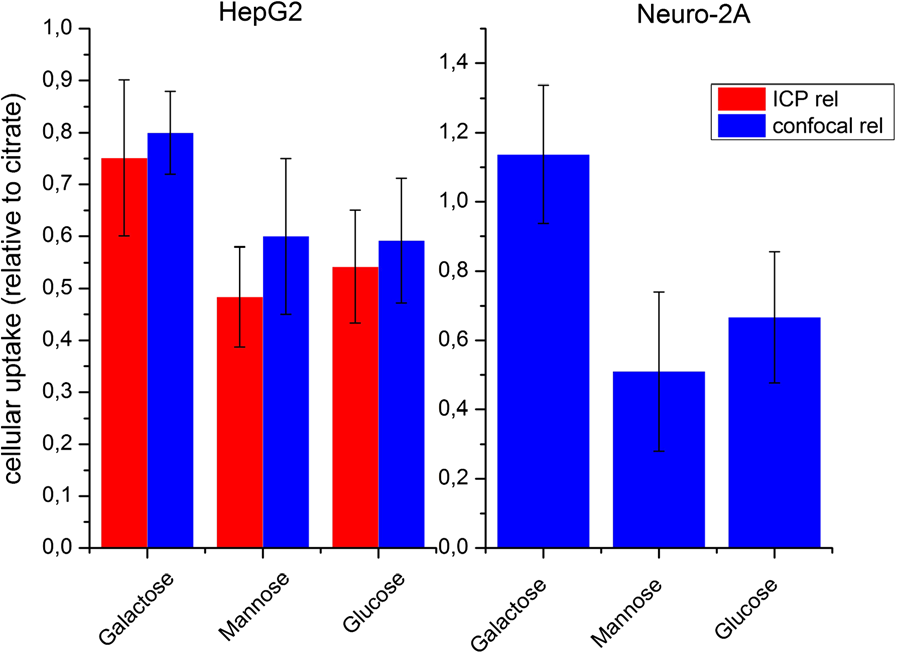
**Cellular uptake of silver nanoparticles with different coatings by in HepG2 cells and Neuro-2A.**

On the other hand, toxicity may also potentially be influenced by different cellular uptake rates. Here, a Trojan-horse mechanism has been often discussed in literature as a responsible for toxicity of silver nanoparticles. According to this, nanoparticles represent carrier vehicles which penetrate into cells, and then release toxic silver ions by dissolution [26]. To get further insights on main factors leading to toxicity, we analyzed the cellular uptake of the different functionalized silver NPs by ICP-MS and by confocal microscopy. ICP-MS and confocal microscopy showed for both cell lines that the less toxic galactose-functionalized nanoparticles are taken up even more efficiently compared to mannose- or glucose- functionalized particles (Figure 4). Moreover, although mannose and glucose-functionalized nanoparticles present similar cellular uptakes, observed toxicities were considerably different. Thus, particles which are largely internalized into cells do not necessarily present the highest toxicity. Actually, in this study, glucose-capped nanoparticles present the highest toxicity as well as protein carbonylation, despite their moderate cellular uptake, compared with other nanoparticles. Interestingly, Vaseem et al. showed that glucose reduces the toxicity of nickel nanoparticles towards A549 cells [27]. Thus, intracellular oxidative stress depending on particles coating was the deciding factor leading to toxicity.

**Figure 5:**
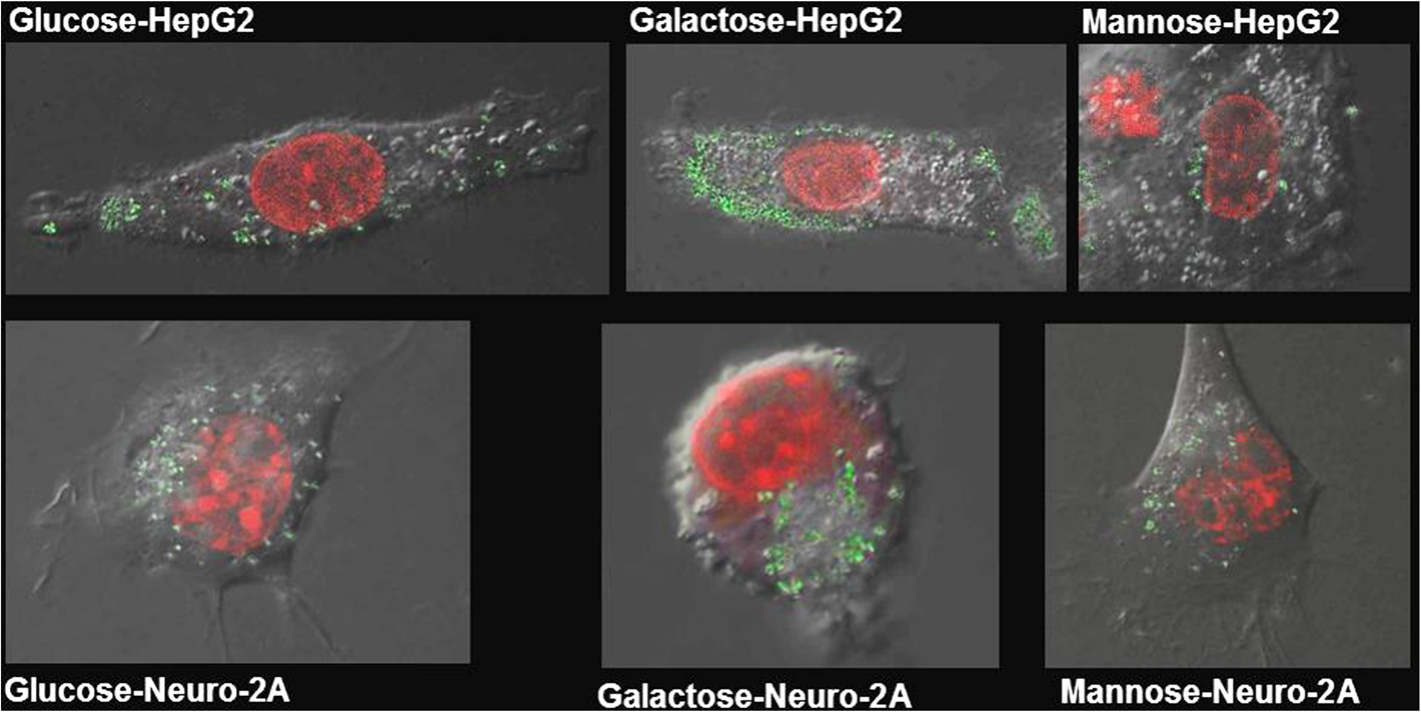
**Confocal microscopy images of cells incubated with prepared nanoparticles.** Cell nuclei are stained in red. Green dots represent fluorescently labelled nanoparticles.

Confocal microscopy images show that cellular localization in Neuro-2A cells for the galactose-coated particles are mainly clustered inside the cytoplasm. Therefore, most likely they are contained inside vesicular structures, such as endosomes or lysosomes. Nevertheless, they apparently do not enter the nucleus (Figure 5). Higher density of particles clusters were observed on one side of each cell. Interestingly, for mannose- and glucose-functionalized particles, clusters seem to be spread more evenly through the cell and intracellular clusters tend to be smaller than particles with other functionalities.

Uptake of nanoparticles depending on surface charge has been discussed by other authors. For instance, Badawy et al. showed that negatively charged silver nanoparticles did not overcome electrostatic repulsion barrier towards similar charged bacillus species [28]. As a result, highly negatively charged citrate silver nanoparticles induced less toxicity than H_2_-Ag nanoparticles. In our case, we also observe a similar correlation between uptake and surface charge for mannose, glucose or EG3 coated Ag-NP, which are more negatively charged and taken up less efficiently. This is consistent with the fact that cells lines used here are also negatively charged due to various carbohydrate moieties. However, in our case, different uptake rates are not related to different toxicities as actually galactose coated NP, which are taken up most efficiently show least toxicity. Eventually this highly increase uptake for galactose coated NP is due to a specific galactose receptor on the surface of cells [23,29].

In fact, internalization of the prepared nanoparticles may depend on various factors, one being surface charge of nanoparticles, another one being the presence of specific receptors on cell surface and finally, it will also depend on the composition of the protein corona [30-32]. We measured the zeta potential of nanoparticles after incubation, showing similar overall negative charges for all particles, which confirmed the formation of the protein corona. Although in the last years, more efforts were invested in order to elucidate the detailed composition of the protein corona, this still remains a challenging question which needs exhaustive analysis and techniques. Nevertheless, based on previous studies, the composition of the protein corona on the surface of functionalized particles presented here is expected to be different depending on the particle coating [33-35].

## Conclusions

Functionalisation of silver nanoparticles with monosaccharides modulates their cellular uptake and toxicity. Galactose and mannose-coated nanoparticles were considerably less toxic to both neuronal-like cells Neuro-2A and hepatocytes, compared to particles functionalized with glucose, ethylene glycol or citrate. Observed toxicity was strongly correlated with intracellular oxidative stress, measured as protein carbonylation, but not to cellular uptake. Summarising, a clear correlation between particle coating, oxidative stress and toxicity has been shown. These results open new perspectives to modulate the bioactivity of Ag-NPs by using carbohydrates.

## Additional file

Additional file 1:
**DLS results/TEM histogram/Calibration curve for sugar quantification/ICP-MS conditions/Carbonyl formation at different concentrations/Extracellular quantification of silver ions.**

## References

[1] Gao J, Gu H, Xu B (2009). Multifunctional magnetic nanoparticles: design, synthesis, and biomedical applications. Acc Chem Res.

[2] Lai C-H, Chang T-C, Chuang Y-J, Tzou D-L, Lin C-C (2013). Stepwise orthogonal click chemistry toward fabrication of paclitaxel/galactose functionalized fluorescent nanoparticles for HepG2 cell targeting and delivery. Bioconjug Chem.

[3] Reidy B, Haase A, Luch A, Dawson K, Lynch I (2013). Mechanisms of silver nanoparticle release, transformation and toxicity: a critical review of current knowledge and recommendations for future studies and applications. Materials.

[4] Sun TY, Gottschalk F, Hungerbühler K, Nowack B (2014). Comprehensive probabilistic modelling of environmental emissions of engineered nanomaterials. Environ Pollut.

[5] Pastoriza-Santos I, Liz-Marzan LM (2008). Colloidal silver nanoplates: state of the art and future challenges. J Mater Chem.

[6] Marradi M, Chiodo F, Garcia I, Penades S (2013). Glyconanoparticles as multifunctional and multimodal carbohydrate systems. Chem Soc Rev.

[7] Lai C-H, Lai N-C, Chuang Y-J, Chou F-I, Yang C-M, Lin C-C (2013). Trivalent galactosyl-functionalized mesoporous silica nanoparticles as a target-specific delivery system for boron neutron capture therapy. Nanoscale.

[8] Chiodo F, Marradi M, Calvo J, Yuste E, Penadés S (2014). Glycosystems in nanotechnology: gold glyconanoparticles as carrier for anti-HIV prodrugs. Beilstein J Org Chem.

[9] Zhang C, Wan X, Zheng X, Shao X, Liu Q, Zhang Q, Qian Y (2014). Dual-functional nanoparticles targeting amyloid plaques in the brains of Alzheimer's disease mice. Biomaterials.

[10] Farr TD, Lai C-H, Grünstein D, Orts-Gil G, Wang C-C, Boehm-Sturm P, Seeberger PH, Harms C (2014). Imaging early endothelial inflammation following stroke by core shell silica superparamagnetic glyconanoparticles that target selectin. Nano Lett.

[11] Seeberger PH, Werz DB (2007). Synthesis and medical applications of oligosaccharides. Nature.

[12] Mammen M, Choi S-K, Whitesides GM (1998). Polyvalent interactions in biological systems: implications for design and use of multivalent ligands and inhibitors. Angew Chem Int Ed.

[13] Yin Win K, Feng S-S (2005). Effects of particle size and surface coating on cellular uptake of polymeric nanoparticles for oral delivery of anticancer drugs. Biomaterials.

[14] Chithrani BD, Chan WCW (2007). Elucidating the mechanism of cellular uptake and removal of protein-coated gold nanoparticles of different sizes and shapes. Nano Lett.

[15] Miao A-J, Schwehr KA, Xu C, Zhang S-J, Luo Z, Quigg A, Santschi PH (2009). The algal toxicity of silver engineered nanoparticles and detoxification by exopolymeric substances. Environ Pollut.

[16] Sur I, Cam D, Kahraman M, Baysal A, Culha M (2010). Interaction of multi-functional silver nanoparticles with living cells. Nanotechnology.

[17] Johnston HJ, Hutchison G, Christensen FM, Peters S, Hankin S, Stone V (2010). A review of the in vivo and in vitro toxicity of silver and gold particulates: particle attributes and biological mechanisms responsible for the observed toxicity. Crit Rev Toxicol.

[18] Pillai ZS, Kamat PV (2003). What factors control the size and shape of silver nanoparticles in the citrate ion reduction method?. J Phys Chem B.

[19] Woehrle GH, Hutchison JE, Özkâr S, Finke RG (2006). Analysis of nanoparticle transmission electron microscopy data using a public- domain image-processing program, image. Turk J Chem.

[20] Kittler S, Greulich C, Gebauer JS, Diendorf J, Treuel L, Ruiz L, Gonzalez-Calbet JM, Vallet-Regi M, Zellner R, Köller M, Epple M (2010). The influence of proteins on the dispersability and cell-biological activity of silver nanoparticles. J Mater Chem.

[21] Drescher D, Orts-Gil G, Laube G, Natte K, Veh RW, Oesterle W, Kneipp J (2011). Toxicity of amorphous silica nanoparticles on eukaryotic cell model is determined by particle agglomeration and serum protein adsorption effects. Anal Bioanal Chem.

[22] Orts-Gil G, Natte K, Drescher D, Bresch H, Mantion A, Kneipp J, Österle W (2011). Characterisation of silica nanoparticles prior to in vitro studies: from primary particles to agglomerates. J Nanoparticle Res.

[23] Lai C-H, Lin C-Y, Wu H-T, Chan H-S, Chuang Y-J, Chen C-T, Lin C-C (2010). Galactose encapsulated multifunctional nanoparticle for HepG2 cell internalization. Adv Funct Mater.

[24] Kittler S, Greulich C, Diendorf J, Köller M, Epple M (2010). Toxicity of silver nanoparticles increases during storage because of slow dissolution under release of silver ions. Chem Mater.

[25] Loza K, Diendorf J, Sengstock C, Ruiz-Gonzalez L, Gonzalez-Calbet JM, Vallet-Regi M, Koller M, Epple M (2014). The dissolution and biological effects of silver nanoparticles in biological media. J Mater Chem B.

[26] Park E-J, Yi J, Kim Y, Choi K, Park K (2010). Silver nanoparticles induce cytotoxicity by a Trojan-horse type mechanism. Toxicol in Vitro.

[27] Vaseem M, Tripathy N, Khang G, Hahn Y-B (2013). Green chemistry of glucose-capped ferromagnetic hcp-nickel nanoparticles and their reduced toxicity. RSC Adv.

[28] El Badawy AM, Silva RG, Morris B, Scheckel KG, Suidan MT, Tolaymat TM (2010). Surface charge-dependent toxicity of silver nanoparticles. Environ Sci Technol.

[29] Barondes S, Rosen S (1976). Cell surface carbohydrate-binding proteins: role in cell recognition. Neuronal Recognit Springer US.

[30] Petri-Fink A, Steitz B, Finka A, Salaklang J, Hofmann H (2008). Effect of cell media on polymer coated superparamagnetic iron oxide nanoparticles (SPIONs): colloidal stability, cytotoxicity, and cellular uptake studies. Eur J Pharm Biopharm.

[31] Bajaj A, Samanta B, Yan H, Jerry DJ, Rotello VM (2009). Stability, toxicity and differential cellular uptake of protein passivated-Fe3O4nanoparticles. J Mater Chem.

[32] Lesniak A, Fenaroli F, Monopoli MP, Äberg C, Dawson KA, Salvati A (2012). Effects of the presence or absence of a protein corona on silica nanoparticle uptake and impact on cells. ACS Nano.

[33] Dorota Walczyk FBB, Monopoli MP, Lynch I, Dawson KA (2010). What the cell “sees” in bionanoscience. J Am Chem Soc.

[34] Lynch I, Cedervall T, Lundqvist M, Cabaleiro-Lago C, Linse S, Dawson KA (2007). The nanoparticle-protein complex as a biological entity; a complex fluids and surface science challenge for the 21st century. Adv Colloid Interf Sci.

[35] Monopoli M, Walczyk D, Campbell A, Elia G, Lynch I, Baldelli Bombelli F, Dawson K (2011). Physical-chemical aspects of protein corona: relevance to in vitro and in vivo biological impacts of nanoparticles. J Am Chem Soc.

