# Supplementary material for: Carbohydrate functionalization of silver nanoparticles modulates cytotoxicity and cellular uptake
Source: J Nanobiotechnology. 2014 Dec 19;12:59. doi: 10.1186/s12951-014-0059-z (PMC4275941; doi:10.1186/s12951-014-0059-z)
Supplement: Additional file 1: — DLS results/TEM histogram/Calibration curve for sugar quantification/ICP-MS conditions/Carbonyl formation at different concentrations/Extracellular quantification of silver ions. [file 12951_2014_59_MOESM1_ESM.docx]

**Supplementary Information (SI)**

| **sample** | **Size DLS (nm)** |  | | **PDI DLS (‰)** | |  | | **ZP (-mV)** | |  | **Sugar loading** | | |  |  |
| --- | --- | --- | --- | --- | --- | --- | --- | --- | --- | --- | --- | --- | --- | --- | --- |
|  | **water** | **CCM** | | **water** | | **CCM** | | **water** | | **CCM*** | **(molecule/nm^2^)** | | |  |  |
|  |  |  | |  | |  | |  | |  | | |  | | |
| Ag- Citrate | 126 ± 2 | 94 ± 2 | 0,46 | | 0,24 | | 35 ± 4 | | - | | |  | | |  |
| Ag- Glucose | - | 91 ± 3 | - | | 0,29 | | 21 ± 4 | | 8 ± 2 | | | 3,2 ± 1,5 | | |  |
| Ag-Mannose | 95 ± 3 | 80 ±3 | 0,44 | | 0,33 | | 19 ± 3 | | - | | | 3,5 ± 1,4 | | |  |
| Ag-EG3 | 128 ± 3 | 93 ±3 | 0,42 | | 0,26 | | 13 ± 2 | | 7 ± 2 | | | - | | |  |
| Ag-Galactose | 83 ± 2 | 76 ± 2 | 0,39 | | 0,29 | | 23 ± 2 | | 6 ± 2 | | | 3,9 ± 1,0 | | |  |

* Without purification, potential correspond to excess of proteins

S.1: PC Characterization of nanoparticles (concentration 10 µg/L).





S.2: histogram of particles sized by TEM

S3: Example of calibration curve for the quantification of sugar loading.

| Plasma power | 1550 W |
| --- | --- |
| Cooling gas flow | 14 L min^-1^ |
| Auxiliary gas flow | 0.8 L min^-1^ |
| Nebulizer gas flow | 0.8 L min^-1^ |
| Detected isotopes | ^107^Ag, ^109^Ag, ^139^La |
| Dwell time | 10 ms |
| Spray chamber cooling | 3 °C |

S.4: Experimental parameters for ICP-MS (iCAP-Q)





S.5: Production of protein carbonyls at different particles concentrations. AgGlu displayed strongest effects and induced protein carbonylation already at 2.5 pM. Furthermore also AgCit induced protein carbonylation, but only at 5 pM while at lower doses no effect was detected. For AgGlu and AgCit the increase in protein carbonylation was statistically significant. AgMan and AgEG3 could slightly induce carbonylation, but effects were not as pronounced as for AgCit and AgGlu and were statistically not significant. AgGal displayed no effect at all tested doses.

S.6: Extracellular quantification of free silver ions by ICP-MS. No silver can be detected, indicating that dissolution of silver nanoparticles in cell culture medium (like in extracellular medium) does not result in free silver ions due to precipitation of AgCl and protein complexes.
